# Antagonistic endophytes rescue ginger from bacterial wilt by enriching the beneficial root-associated bacteria and regulating antibacterial root exudates

**DOI:** 10.3389/fmicb.2026.1890218

**Published:** 2026-07-30

**Authors:** Keke Dang, Wenrui Zhao, Junwei Peng, Yang Sun, Lan Zhang, Yuanhua Dong, Jiangang Li

**Affiliations:** 1 School of Energy, Environmental and Geomatics Engineering, Anqing Normal University, Anqing, China; 2 State Key Laboratory of Soil and Sustainable Agriculture, Institute of Soil Science, Chinese Academy of Sciences, Nanjing, China; 3 University of Chinese Academy of Sciences, Beijing, China; 4 Nanjing Institute of Environmental Sciences, Ministry of Ecology and Environment, Nanjing, China

**Keywords:** antagonistic endophytes, bacterial community, ginger, *Ralstonia solanacearum*, root exudates

## Abstract

**Introduction:**

Bacterial wilt of ginger, caused by *Ralstonia solanacearum* (RS), significantly impacts ginger production. Probiotics, such as antagonistic bacteria, offer a safe and effective approach to disease control.

**Methods:**

This study investigated the effects of two antagonistic endophytes (AEs), *Enterobacter asburiae* PA16 and *Pseudomonas indica* PA2457, on the ginger root-associated bacteria and exudates in the presence of RS infection by high-throughput sequencing (HTS) and gas chromatography–mass spectrometry (GC–MS).

**Results and discussion:**

The results showed that AEs promoted plant health, enhanced the niche breadth and stability of rhizobacteria, increased plant beneficial groups such as *Mesorhizobium*, *Massilia*, and *Bosea*, while decreased the abundance of genus *Ralstonia* in which the pathogenic bacteria RS resides. In the term of root exudates, up-regulated Gibberellin and Trehalose after AEs inoculation, while the control treatment enriched more antibacterial such as Isovanillic acid, Arbutin, and Sclareolide. Overall, the health and growth of ginger were enhanced through the enrichment of root-related beneficial bacteria and exudates stimulated by AEs. These findings offer strong support for the effective using AEs in the management of ginger bacterial wilt.

## 1 Introduction

Bacterial wilt is a soil-borne disease caused by *Ralstonia solanacearum* (RS), which poses a significant threat to crops cultivation such as ginger, tomato, and tobacco, leading to substantial losses (Jiang et al., 2017). Once the RS infects plant roots, it will quickly sweep into the stems, damage the microbial community inside plants and alter the rhizosphere microenvironment, expediting the plant to wither and die (Tao et al., 2024). Plant microbiomes and root exudates have been reported as essential carriers for material cycling, energy exchange, and information transfer between the plants and the external environment (Han et al., 2025; Ma et al., 2022; Carrión et al., 2019). In contrast to the environmental problems caused by chemical pesticides, plant microbiome plays a multifaceted role in suppressing diseases, increasing yields, aiding in damage repair, and promoting plant growth (Iqbal, 2026; van der Lelie et al., 2009; Carrión et al., 2019). To harness more effective microbial resources for plant disease management, researchers are now exploring untapped microbes from specific environmental niches, such as plant endophytic environments (Chandwani et al., 2023; Wang et al., 2019).

Microbes that spent all or part of their life cycle in plant tissues are called endophytes. Similar to the human intestinal microbiota, endophytic bacteria are non-pathogenic and can coexist with the host, thereby improving plant fitness or protecting it from pathogenic infections and contributing to host health (Tao et al., 2024). Pathogen invasion is considered one of the most important biotic stress factors affecting the assembly of plant microbiota and the composition of root exudates. With the invasion of pathogen, plant bacteria were altered in abundance, niche breadth and community stability, leading to a reduced environmental resilience (Ma et al., 2026; Umana et al., 2015). Studies have suggested that antagonistic endophytes (AEs) can reshape the composition and interactions of indigenous bacteria, which help plants to enhance their ability to cope with disease (Liu et al., 2021). For example, wider habitat niche breadths, stronger stability, greater robustness, and stronger competition of bacteria exhibit higher environmental tolerances among the bacterial community (Peng et al., 2025; Farjalla et al., 2012; Coyte et al., 2015). However, our understanding of whether the plant AEs employs the strategies for optimizing the microenvironment to make ingenious use of microbial benefits upon pathogen infection remains largely unknown.

Recent studies have suggested that these beneficial endobacteria such as *Pseudomonas* spp., *Streptomycetes lavendulae*, *Bacillus subtilis*, *Bacillus cereus* exhibit significant inhibitory effects on RS, thereby reducing the mortality rate associated with ginger blast (Chandwani et al., 2023; Wang et al., 2019; Costa et al., 2006). Additionally, *Enterobacter*, a newly discovered endophytic bacterial strain isolated from ginger tissue, constituted 86.76% of the stable simplified bacterial community, inhibiting pathogens while maintaining bacterial community homeostasis of plants (Roy et al., 2019; Liu et al., 2019). Such beneficial AEs are considered to be vital for maintaining plant health. Regarding the protective mechanism of AEs, previous studies have summarized it into two aspects: direct microbe–pathogen interactions and indirect plant-mediated protection (Tao et al., 2024). On the one hand, AEs can kill the pathogens directly by niche competition or secondary metabolites toxicity, which is well-known for researchers. On the other hand, AEs may suppress and prevent plant diseases indirectly via regulating root exudates. Root exudates have garnered increased attention in recent years due to their crucial role in plant-microbe interactions. These exudates consist of a variety of chemicals released by plant roots or endobacteria into the surrounding soil, including inorganic small molecules, carbohydrates, amino acids, and high-molecular substances such as polysaccharides and proteins (Lebeis et al., 2015). In response to unfavorable environmental conditions like pathogen invasion, extreme temperatures, high salinity, or drought, plants adjust their root secretion behavior to sustain their normal physiological functions. This adjustment involves altering in the type and quantity of root exudates, representing a significant plant response to challenging environments (Uribe-Acosta et al., 2025; Chang et al., 2014; Rho et al., 2018). Furthermore, plants can modulate the rhizosphere and root endospheric bacteria by changing the type and composition of exudates. For instance, wheat and corn can release benzoxazine compounds to reshape root-associated microbial communities and boost plant defense mechanisms (Hu et al., 2018). However, the potential mechanisms underlying the mutualistic interactions between AEs and plants bacteria/root exudates are still poorly understood.

In present study, two AEs, previously isolated from healthy ginger root, *Enterobacter asburiae* PA16 and *Pseudomonas indica* PA2457, were used as biocontrol agents (Dang et al., 2023). Using bacterial amplicon sequencing, gas chromatography–mass spectrometry (GC–MS), and culture based methods, we aim to elucidate the changes of bacterial community and exudates in the ginger root under RS invasion with AEs addition, and investigate the comprehensive impact of AEs on plant growth and health. We hypothesized that the colonization of AEs reduces the incidence of diseases and optimizes the composition and structure of the bacterial community and root exudates. We investigated the effects of AEs on the microenvironment of ginger roots, and clarified the biological control mechanisms of antagonistic endophytes, providing suggestions for green and efficient control of ginger bacterial wilt.

## 2 Methods

### 2.1 Antagonistic endophytes trains and their cultivation conditions

Two strains of AEs, *Enterobacter asburiae* PA16 and *Pseudomonas indica* PA2457, were isolated from the roots of healthy *Zingiber officinale* (ginger) in our previous research (Dang et al., 2023). Their inhibitory activities against *Ralstonia solanacearum* has been confirmed, and they have been characterized using physiological, biochemical, and molecular biology techniques, including the methyl methanol test, methyl red test, ornithine decarboxylase, lysine decarboxylase, D-xylose, L-arabinose, lactose, sorbitol, sucrose, and citrate utilization refers to the Common Bacterial System Identification Manual and the Berger Bacterial Identification Manual (Buchanan and Gibbons, 1994; Supplementary Table S1). Additionally, multiple sequence homology analysis was performed using MEGA-6 software to construct a phylogenetic tree. Based on the identification results of the antagonistic bacteria, which were identified as belonging to *Enterobacter asburiae* and *Pseudomonas indica* (Supplementary Figure S1). The isolation and purification of the antagonistic endophytes were cultured on nutrient agar (NA) culture medium consisting of 10 g of glucose, 5 g of peptone, 3 g of beef extract, 0.5 g of yeast powder, and 1 L of deionized water, with the addition of 18 g of agar powder for solid medium preparation.

### 2.2 Pot experiment

The soil used was collected from Yingtan red soil ecological experiment station, Chinese Academy of Sciences in Yujiang District, Yingtan City, Jiangxi Province, China (28°20’N, 116°93′E), in April 2023. The soil nutrient contents were as follows: total nitrogen (N): 0.04%; total phosphorus (P): 0.03%; total potassium (K): 1.23%; available N: 13.37 mg/kg; available P: 5.93 mg/kg; available K: 76.82 mg/kg. Before conducting the pot experiment, in April 2023, grind and sieve the soil (5 mm) and add a certain amount of chemical fertilizer and mix well to ensure the nutrition of the ginger seedlings: N 0.15 g/kg (urea), P_2_O_5_ 0.2 g/kg (calcium magnesium phosphate fertilizer) and K_2_O 0.20 g/kg (potassium chloride) were added to potted soil to ensure the nutritional requirements of ginger.

**Figure 1:**
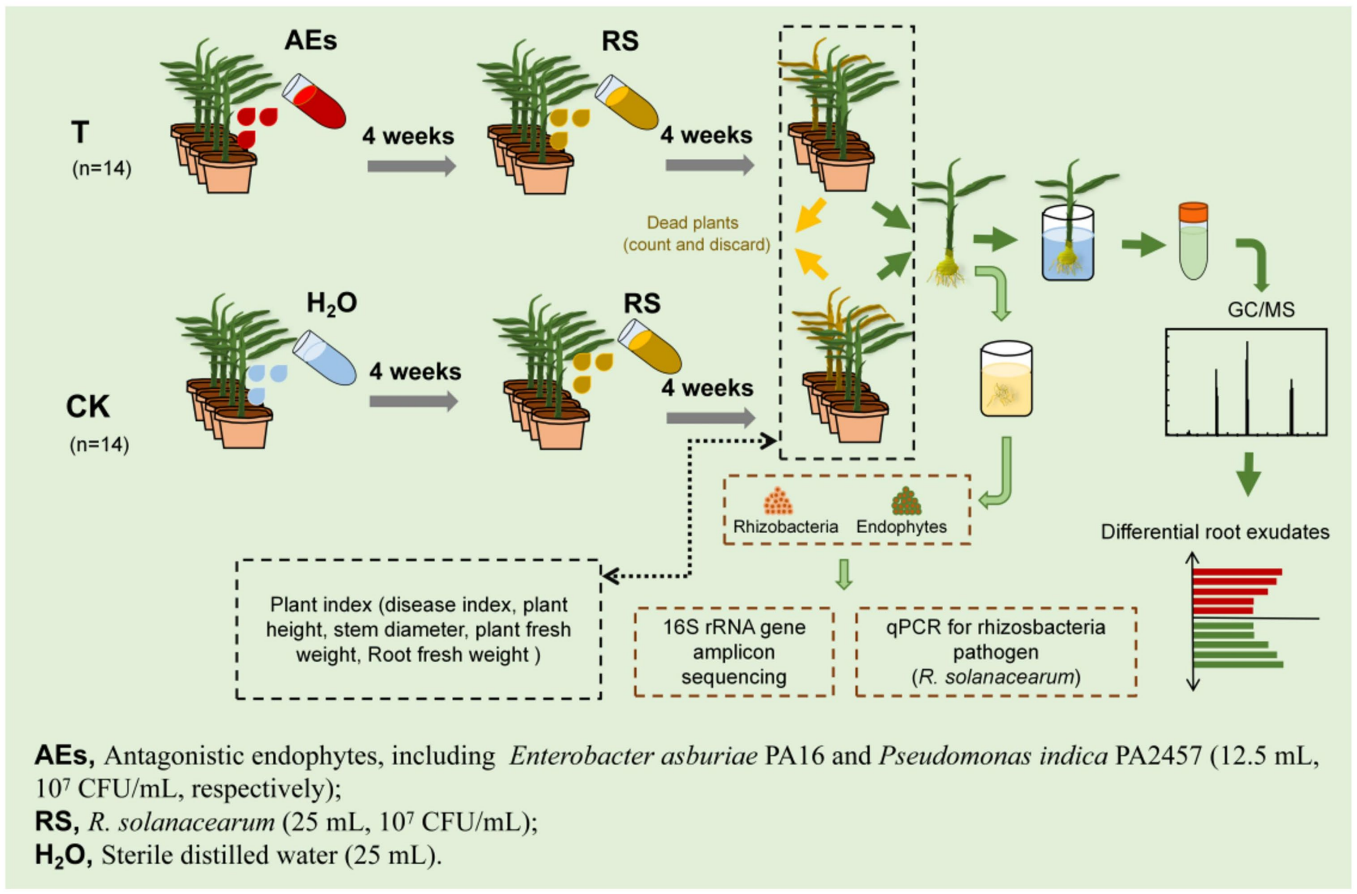
Schematic diagram of experimental design.

The potted experiment was conducted in a greenhouse with the temperature at 28 °C, humidity at 60%, and natural light. A total of 28 pots were set up, and 1.0 kg of the above-mentioned air-dried soil samples were placed into each pot. The germinated ginger seedlings (5 cm high, one plant per pot) with uniform growth were transplanted into pots. After cultivating for 14 days, 14 pots were randomly selected as treatment (T), and the other 14 pots were marked as control (CK). T treatment was inoculated of a mixed suspension of 12.5 mL suspension of *Enterobacter asburiae* PA16 and 12.5 mL *Pseudomonas indica* PA2457 both with the density of 10^7^ CFU/mL. Simultaneously, CK was treated with the same volume of sterile water (25 mL). Twenty-eight days after transplanting, roots were irrigated with RS suspension (25 mL, 10^7^ CFU/mL) both in treatments of CK and T (Figure 1).

The plant height, stem diameter, disease incidence, and RS density were measured 14 days after inoculation of pathogen. Plant height and stem diameter were measured using a ruler and a vernier caliper, respectively. Disease incidence was calculated by this formula: Incidence rate(%) = total number of infected plants/total number of plants × 100. The pathogen density (RS density) was determined by Quantitative Real−time PCR using specific primers.

### 2.3 Bacteria collection, DNA extraction, and Illumina sequencing

Rhizobacteria and endobacteria were collected when harvested. The collection method is optimized based on the previous researches (Phazna et al., 2020; Wemheuer et al., 2020). Specifically, ginger plants were removed from soil completely and shaken to remove all loosely adhering soil on root surface. Then brushed tightly adhering soil on root surface with a sterile brush, which collected as the rhizosphere soil (storing at −20 °C for DNA extraction later). Disinfect root surfaces before acquiring endobacteria: soak in 70% ethanol for 20 s, 3% sodium hypochlorite for 30 s, 70% ethanol for 20 s, followed by immersion 3 times in distilled sterile water for 10 s. The surface−sterilized roots were subsequently dried on sterile filter paper. Then place the roots into the sterile mortar, 5 mL phosphate buffer (pH = 7) added, ground, and the grinding solution (endobacteria suspension) collected for subsequent DNA extraction.

The DNA samples of rhizosphere and endophytic bacteria were extracted using GenElute™ Bacterial Genomic DNA Kit (Sigma−Aldrich) in accordance with the manufacturer’s protocol. Then quantified the DNA samples extracted with spectrophotometrically (Nanodrop ND − 2000, Thermo Scientific, Waltham, MA, USA). The rhizobacteria 16S rRNA gene was amplified with V4–V5 regions by Polymerase Chain Reaction (PCR) using the primers 515F (5’-GTGCCAGCMGCCGCGGTAA-3′) and 907R (5’-CCGTCAATTCMTTTRAGTTT-3′), while the 16S rRNA genes of endobacteria were amplified with V5–V7 regions by PCR using the universal primers 799F (5’-AACMGGATTAGATACCCKG-3′) and 1193R (5’-ACGTCATCCCCACCTTCC-3′) (Biddle et al., 2008; Horton et al., 2014). The raw sequencing data were further processed using QIIME toolkit (v1.2.1, Caporaso et al., 2010) as follows: keep the quality score greater than 25, sequence length between 200 and 400 bp, no maximum ambiguous bases and mismatched bases in the primer.

### 2.4 Quantitative real−time PCR

Quantitative real−time PCR was performed using TaqMan Universal PCR master mix for RS in rhizosphere. The specific primers of RS were *Rsol-flic*-F (5’-GAACGCCAACGGTGCGAACT-3′) and *Rsol-flic*-R (5’-GGCGGCCTTCAGGGAGGTC-3′) with fragment lengths of 390 bp were used for PCR. PCR reactions were performed with three replicates per treatment using the same DNA extracts used for 454 pyrosequencing. The specific primers of *Enterobacter asburiae* PA16 were EA-2F(5′-CGGAAGCCACGCCTCAAG-3′)and EA-2R (3′-CTGGACAAAGACTGACGCTCAG-5′), and the specific primers of *Pseudomonas indica* PA2457 were PA-1F (5′-CCACCTGGACTGATACTGACAC-3′) and PA-1R (3′-CGCCACTAAGATCTCAAGGATCC-5′). This work is entrusted to Shanghai Lingen Biotechnology Co., Ltd.

### 2.5 Root exudate collection and GC–MS analysis

The collection of root exudates was conducted according to the previous description (Fan et al., 1997). When harvesting, ginger plants were carefully removed from pots, and the soil adhered to the roots was washed off with sterile water followed by three rinses with ultrapure water. The cleaned roots were then placed in small pots with 50 mL of ultrapure water. After 6 h, the extraction of ultrapure aqueous solution (root exudate samples) was collected and stored in a − 80 °C refrigerator for further analysis.

Sample pretreatment prior to GC–MS detection was carried out according to Sun et al. (2020). The root exudates were freeze−dried, and then dissolved in acetonitrile with 1 mL in one sample. Subsequently, 100 μL dissolved solution was transferred to a 2 mL centrifuge tube, and 1 mL of the following mixed solution added: acetonitrile, isopropanol, and water at a 3:3:2 (v/v) ratio. The mixed solution was shaken and mixed for 1 min, then keep at 4 °C for 4 min. The mixture was then vortexed for 3 min. The above steps were repeated twice, and then the mixture was centrifuged at 10,000 g at 4 °C for 10 min. The clear supernatant extract is retained for next analysis. In addition, 50 μL supernatant was taken from each sample and mixed with the quality control sample, respectively. The remaining supernatant was concentrated to dryness. Then, 80 μL methoxy−aminopyridine solution (20 mg/mL) added to dissolve the dried sample, vortex for 1 min, and react at 80 °C for 30 min. Finally, 100 μL BSTFA reagent (containing 1% TMCS, v/v) was added to the sample, and keep 70 °C for 120 min. The sample was then centrifuged at 10,000 g for 4 min, and the clean supernatant was transferred to the injection bottle for GC − TOF MS detection.

### 2.6 Identification of plant-beneficial bacteria (PBB)

The functions of PBB include biocontrol efficiency, plant-growth-promoting and stress resistance were identified based on a PBB database,^1^ which comprised 396 bacterial genera from 17 phyla, 27 classes, 76 orders and 135 families (Li et al., 2023). PBB provide multiple benefits to plants, which can be divided into three major categories: biocontrol capacity (including plant disease suppression and resistance induction), plant-growth-promoting behavior (such as the ability to fix nitrogen, solubilize phosphorus and potassium or produce siderophores and phytohormones), and stress resistance provision (for example, against floods, drought or increased salinity) (Li et al., 2023).

### 2.7 Statistical analysis

Data was analyzed by analysis of variance (ANOVA) after confirming that it met the assumptions of normality and equal variance, which was executed by the anova function in the stats package of R (v4.3, Vienna, Austria). Principal component analysis (PCA) plots were visualized by PAST software (v4.09, Paleontological Statistics, http://folk.uio.no/ohammer/past/) based on Bray−Curtis distances. Software STAMP (v2.1.3, https://beikolab.cs.dal.ca/software/STAMP) was used for histogram drawing. The specificity and occupancy of Amplicon Sequence Variants (ASVs) were calculated as per Dufrene and Legendre (1997). Potential community functions were predicted by the PICRUSt2 study (Phylogenetic Investigation of Communities by Reconstruction of Unobserved States, https://github.com/picrust/picrust2/releases). Specificity−Occupancy (SPEC−OCCU) were calculated and visualized based on a previous study (Gweon et al., 2021). To examine the stability of bacterial community with antagonistic bacteria addition in rhizosphere and endosphere, the natural connectivity was calculated according to the percentile method (Wu et al., 2010; Shi et al., 2020).

## 3 Results

### 3.1 Antagonistic endophytes inhibit ginger bacterial wilt and promote plant growth

**Figure 2:**
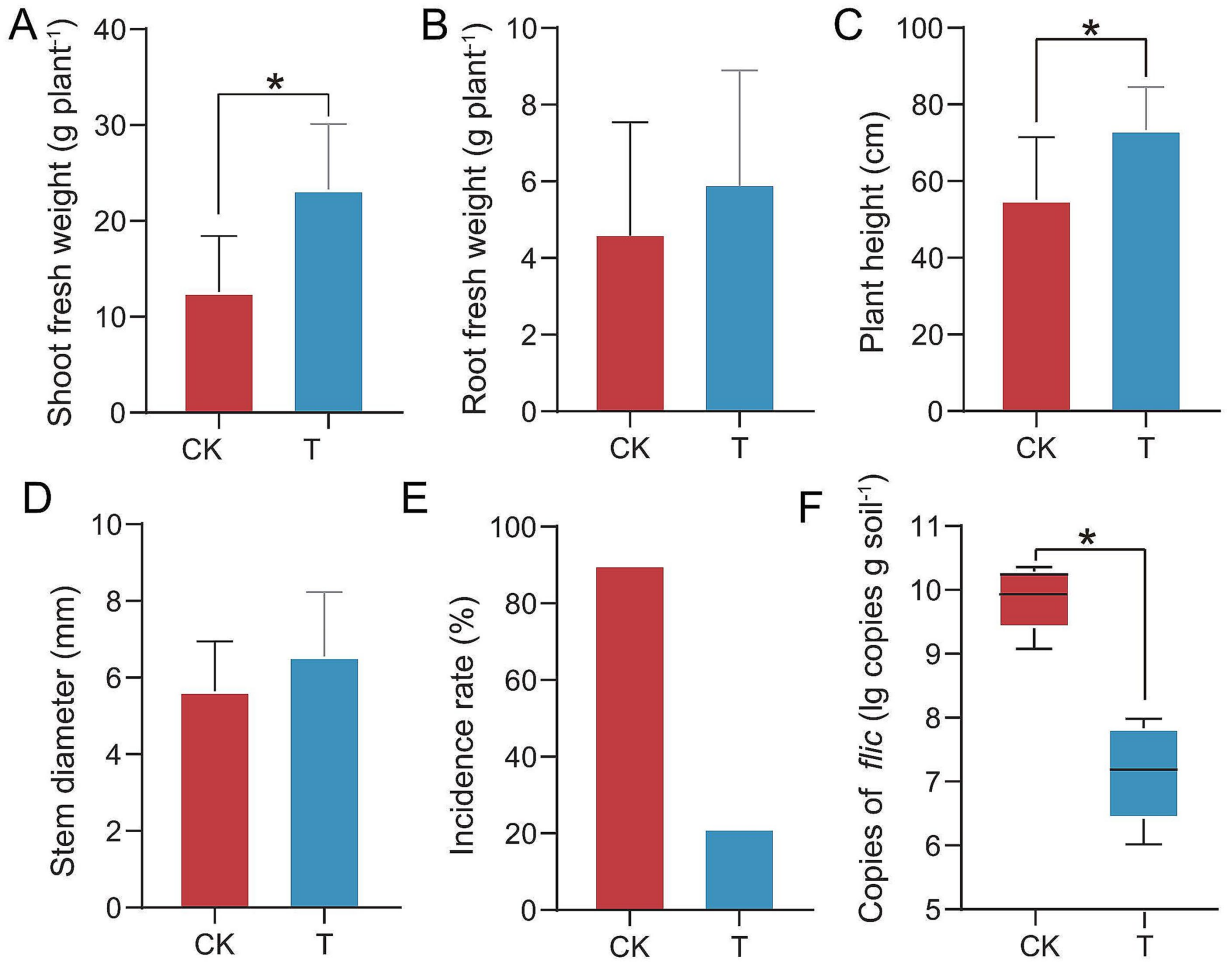
Growth status and the incidence rate of bacterial wilt in ginger with AEs addition. **(A)** Shoot fresh weight; **(B)** Root fresh weight; **(C)** Plant height; **(D)** Stem diameter; **(E)** Incidence rate of bacterial wilt; **(F)** Copies of fliC. Asterisk (*) indicates significant differences between CK and T (*p* < 0.05, t-test).

AEs significantly enhance the growth of ginger. To evaluate the growth status of ginger, key indicators such as fresh weight, plant height, and stem diameter were measured. The results indicated that both shoot fresh weight and plant height were significantly greater in the treatment group (T) compared to the control group (CK) (Figures 2A–D). Specifically, the introduction of antagonistic endophytes resulted in an 85% increase in shoot fresh weight and a 33% increase in plant height. Furthermore, AEs also mitigated bacterial disease occurrence and decreased the density of pathogenic bacteria *R. solanacearum* in the rhizosphere soil. The incidence rate in ginger plants was reduced from 90 to 21% (Figure 2E). Additionally, the number of gene copies of pathogens in CK was more than ten times higher than that of in T (Figure 2F).

### 3.2 Antagonistic endophytes inhibit the growth of pathogen but promote the beneficial bacterial community

Bacterial communities in the endosphere and rhizosphere of ginger were analyzed to investigate the impact of AEs addition on the root-associated bacteria assemblage. After removing adapter-containing reads, a total of 1,007,724 high-quality bacterial 16S rRNA sequences with an average read length of 376 bp were obtained from rhizosphere samples, while 1,037,731 high-quality bacterial 16S rRNA gene sequences with an average read length of 375 bp were obtained from endosphere samples. These sequences were classified into 5,418 and 1,506 bacterial ASVs in rhizosphere and endosphere samples, respectively.

**Figure 3:**
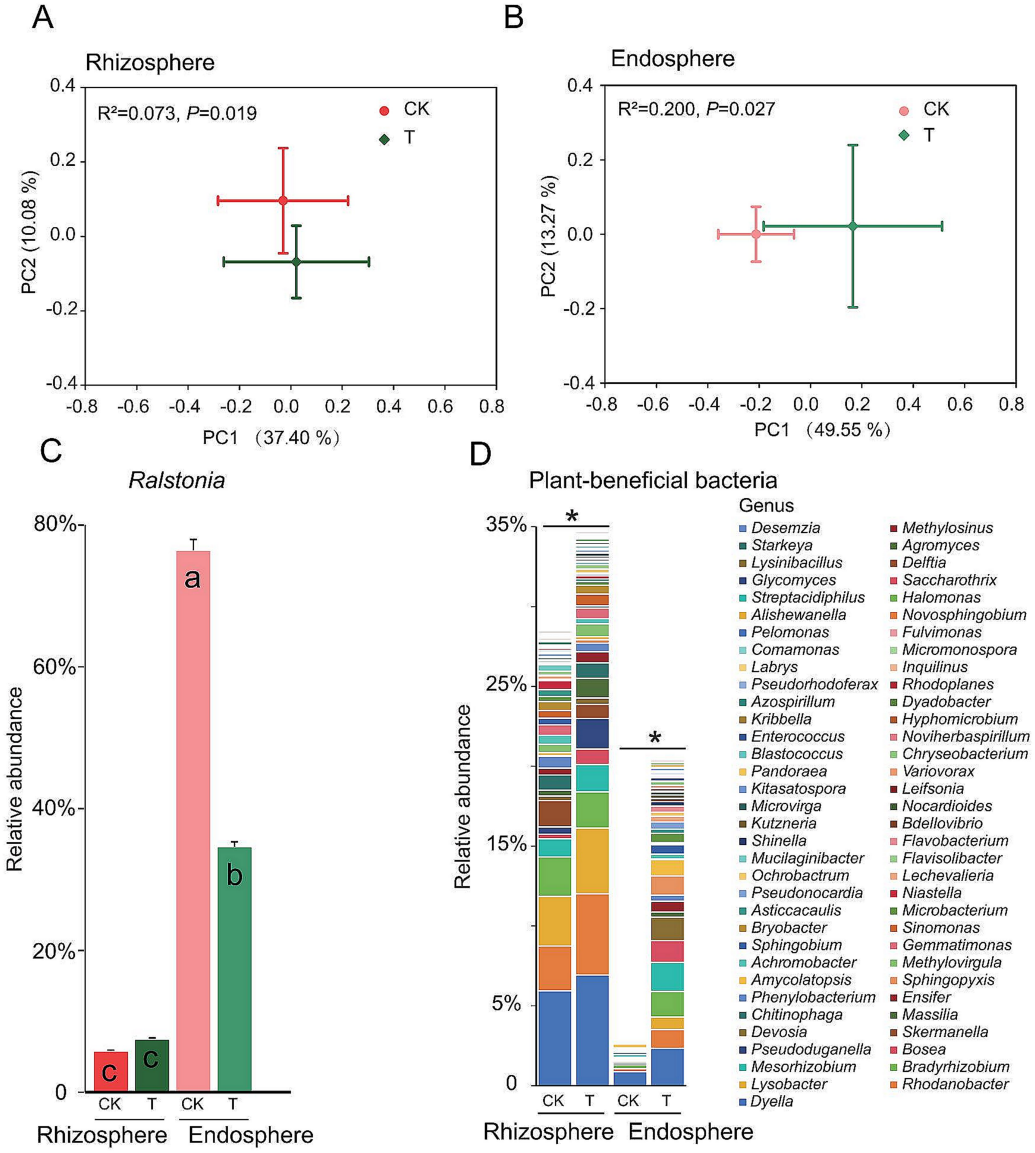
Composition in bacteria communities with AEs addition. Principal component analysis (PCA) of the bacterial community in rhizosphere **(A)** and endosphere **(B)**; **(C)** the relative abundance of *Ralstonia*. Equal letters represent equal averages; different letters represent the statistical differences (*p* < 0.05); **(D)** relative abundance of PBB (at genus level) in different treatment. * *p* < 0.05.

The bacterial community composition was greatly different when AEs were introduced no matter in rhizosphere or endosphere (Figures 3A,B). PERMANOVA analysis and PCA of Bray-Curtis distance showed that the AEs addition exerted a significant effect on both rhizosphere (*R*^2^ = 0.073, *p* = 0.019) and endosphere (*R*^2^ = 0.200, *p* = 0.027). We further investigated the differences at the genus level, it was found that the abundance of *Ralstonia* in the endosphere sample was significantly lower in the T treatment compared to the control treatment (*p* < 0.05, Figure 3C; Supplementary Figure S2), however, there was no significant difference in the rhizosphere soil between the different treatments (Figure 3C; Supplementary Figure S2).

**Figure 4:**
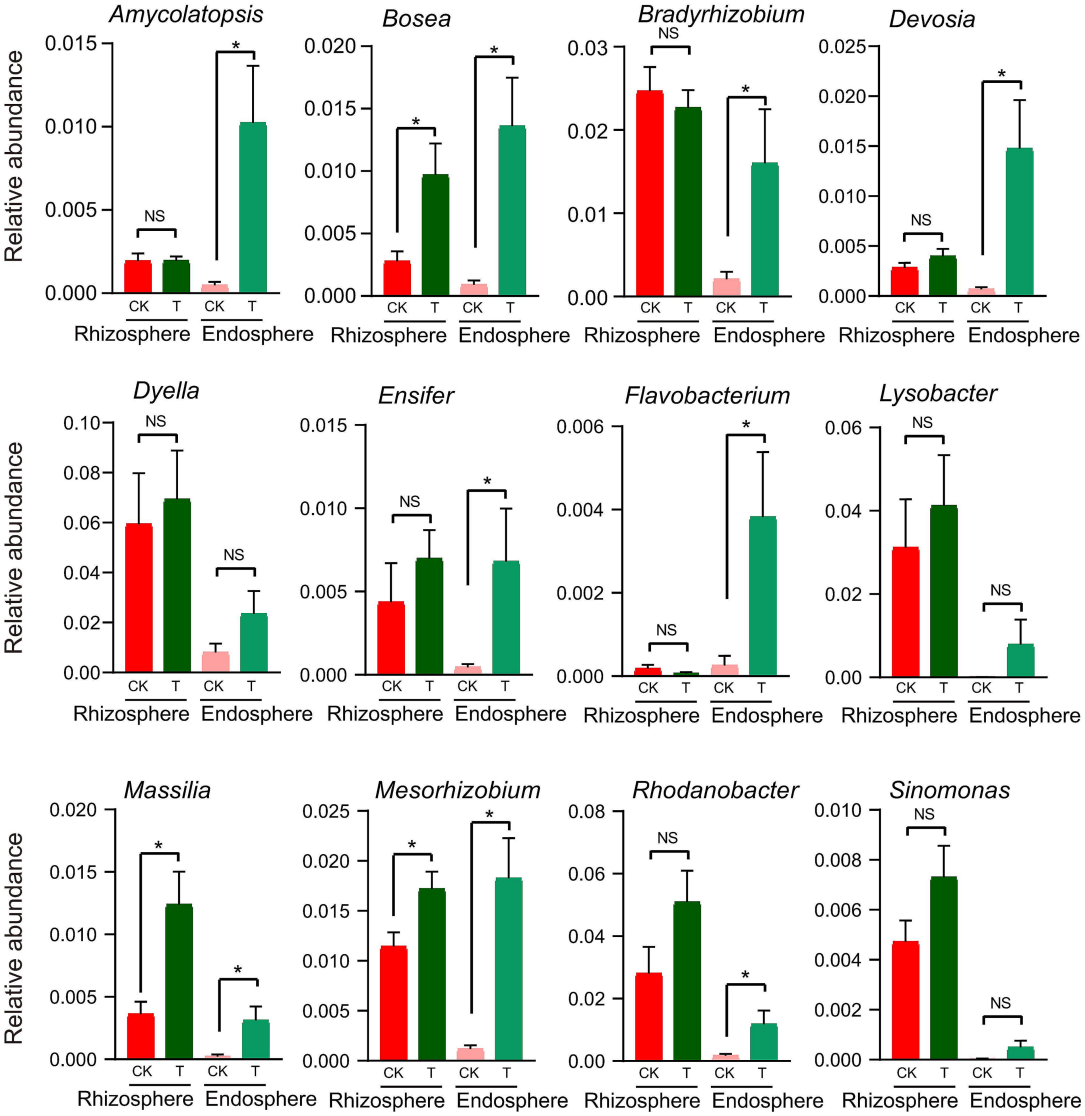
The relative abundance of PBB in different treatment. *p < 0.05.

PBB are essential for promoting plant growth and resilience by engaging in activities such as biological control, nutrient supply, and hormone production. Utilizing a PBB database (Li et al., 2023), we identified 68 genera of PBB in the rhizosphere and endosphere samples, with 15 genera showing an abundance greater than 1%. Furthermore, the abundance of beneficial bacteria is more pronounced in the endosphere than in the rhizosphere (*p* < 0.05, Figure 3D). Interestingly, most of them displayed a higher prevalence in the T treatment compared to CK (Figure 4). Notably, genera like *Mesorhizobium*, *Massilia*, and *Bosea* exhibited a significant advantage in the T treatment both in the rhizosphere and endosphere (Figure 4). Specifically, in the rhizosphere, the T treatment exhibited a 35% abundance of beneficial bacteria, while the CK treatment had 29%. In the endosphere, the T treatment showed a 22% abundance of beneficial bacteria, whereas the CK treatment only had 3% (Figure 3D). Compared with CK, beneficial bacteria such as *Amycolatopsis*, *Bradyrhizobium*, *Devosia*, *Ensifer*, *Flavobacterium*, and *Rhodanobacter* in the endosphere were significantly higher in T treatment (Figure 4).

### 3.3 Antagonistic endophytes alter the ecological niche breadth and enhance the robustness of bacterial network

**Figure 5:**
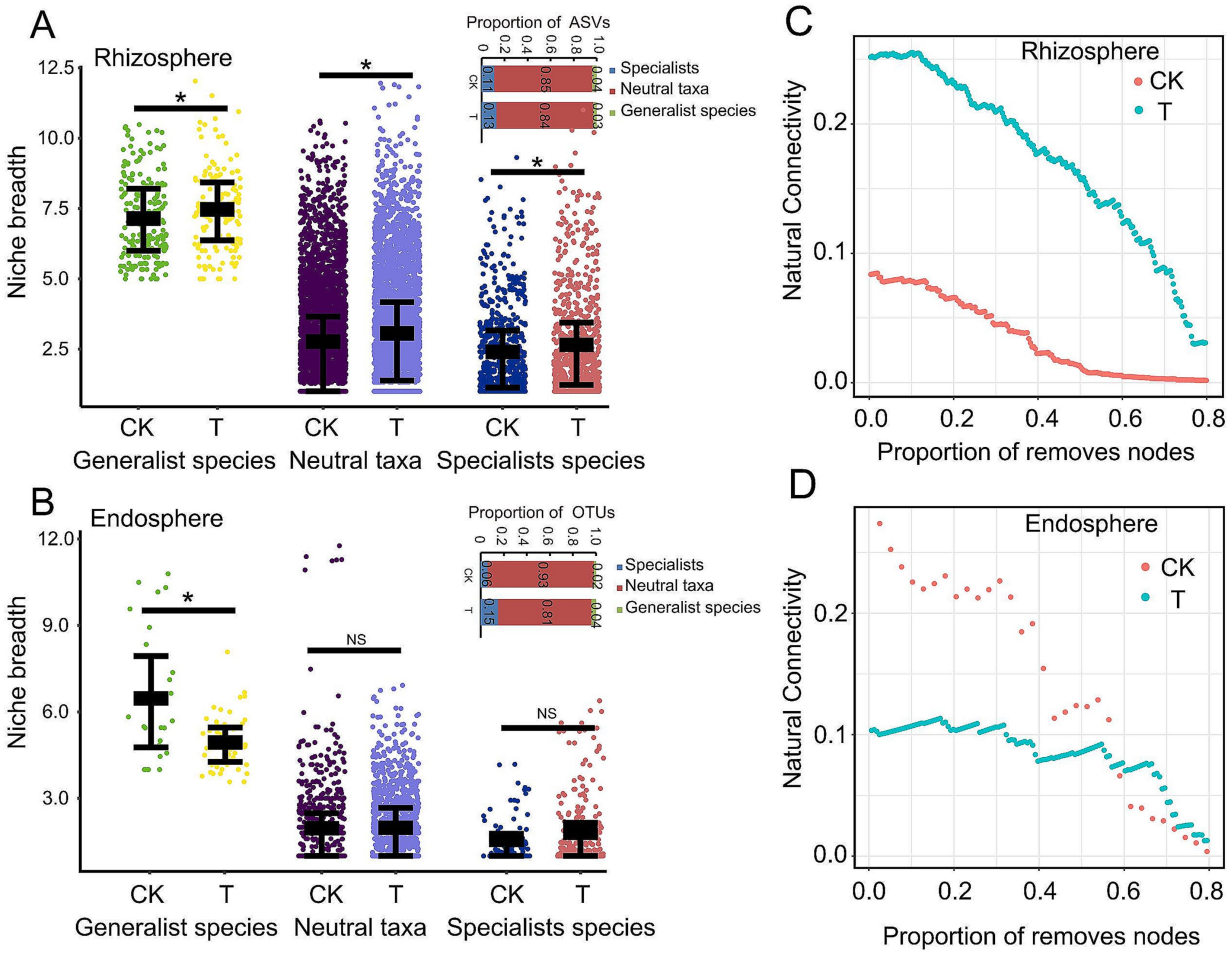
Characteristics of bacterial communities in different treatments. The box plot shows the exponential distribution of the niche breadth of the bacterial communities (generalist species, neutral taxa, and specialists) in rhizosphere **(A)** and endosphere **(B)**. Natural connectivity of bacterial network by treatments in rhizosphere **(C)** and endosphere **(D)**.

To explore the ecological importance of intraspecific variation, we examined the distribution and niche preferences of bacteria in both treatments. Ecological niche breadth refers to the total range of resources utilized by a population within a community. Bacteria (ASV level) were classified into generalist species, neutral taxa, and specialist species based on their niche breadth (Figures 5A,B). In the rhizosphere, the introduction of AEs led to a significant expansion of niche breadth for specialized, neutral, and generalized species (*p* < 0.05, Figure 5A). The niche breadth of generalized species in treatment group (T) was significantly larger than that of the control group (CK) in endoesphere. However, no significant difference was observed between CK and T in terms of neutral taxa and specialist species (Figure 5B). In conclusion, the presence of antagonistic bacteria mainly widened the niche breadth of rhizosphere bacteria.

A natural connectivity analysis was conducted to assess the robustness of bacterial networks in CK and T treatments. The results revealed distinct trends between the two treatments. In the rhizosphere, natural connectivity values were consistently higher in T compared to CK (Figure 5C). However, in the endosphere, CK had higher natural connectivity than T when the number of removal nodes was below 60%. Conversely, when the number of removal nodes exceeded 60%, T exhibited higher natural connectivity than CK (Figure 5D). These findings underscore the substantial impact of antagonistic bacteria on the robustness of the soil bacterial network.

### 3.4 Antagonistic endophytes reshape the abundance and function of root exudates

**Figure 6:**
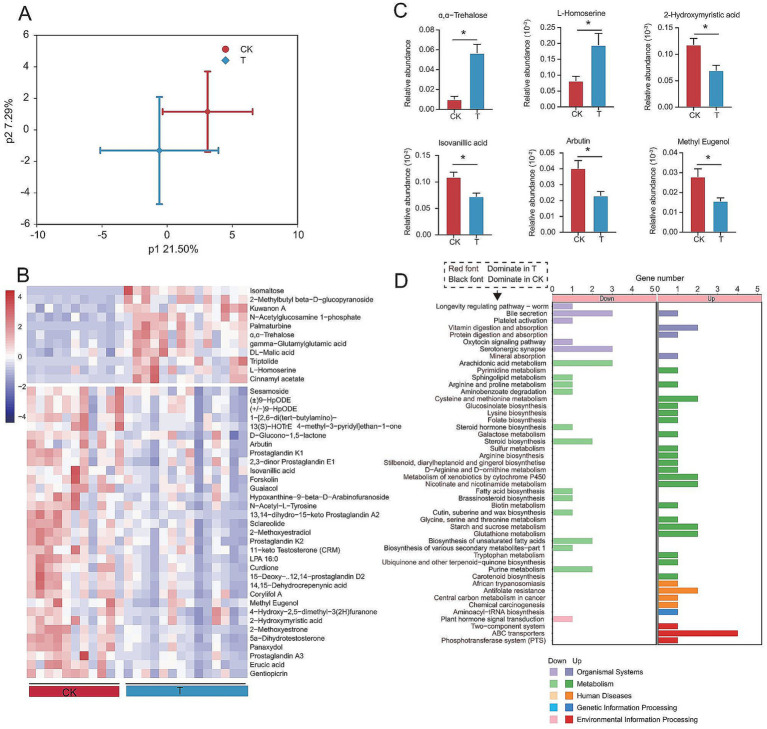
Differences in root exudates after two endophytic bacteria addition. **(A)** Partial Least Squares Discriminant Analysis (PLS-DA) results based on Bray-Curtis distances in root exudates; **(B)** Heat Map illustrating the up-regulated and down-regulated patterns of the root exudates in T compared with CK; Each red block represents a metabolite with higher abundance, while a blue block represents a metabolite with lower abundance; **(C)** The bar chart shows significant different root exudates between the CK and T groups; **(D)** Statistics of the number of differential genes annotated to the pathway. The ordinate represents the metabolic pathway, and the abscissa is the number of differential genes annotated to the pathway. Asterisk (*) indicates significant differences between CK and T (*p* < 0.05, t-test).

Root exudates, a diverse group of carbon compounds, are actively secreted from various parts of the root system into the surrounding soil. These exudates are crucial for maintaining the vitality of the microecology system in the rhizosphere and play a significant role in material cycling within the root microenvironment. A total of 559 metabolites were identified through GC–MS analysis. Partial Least Squares Discriminant Analysis (PLS-DA) revealed distinct differences in root exudate profiles among different treatments (Figure 6A, *R*^2^ = 0.173, *p* = 0.002). Forty-four types of root exudates showed significant differences (*p* < 0.01) between the control (CK) and (T) groups. Specifically, eleven metabolites were significantly enriched in the T group, while thirty-three metabolites were significantly enriched in the CK group (Figure 6B). The addition AEs led to improvements in plant growth regulators such as L-Homoserine and *α*,α-Trehalose. On the other hand, antibacterial metabolites like Isovanillic acid, Arbutin, and Methyl Eugenol increased in the CK treatment (Figure 6C). To elucidate the metabolic pathway influenced by root exudates, we analyzed the changes of gene expression following the introduction of AEs (Figure 6D). Compared to the control (CK), metabolic pathways associated with genetic and environmental information processing showed significant up-regulation, such as aminoacyl-tRNA biosynthesis, two-component system, ATP-binding cassette transporter (ABC transporters), and phosphotransferase system (PTS), while plant hormone signal transduction was down-regulated. Organismal systems exhibited a mixed response with vitamin digestion and absorption, protein digestion and absorption, and mineral absorption showing up-regulation, and bile secretion, platelet activation, oxytocin signaling pathway, and serotonergic synapse showing down-regulation. Moreover, in treatment (T), a majority of metabolic pathways (63.64%) were up-regulated, encompassing processes like mineral absorption, pyrimidine metabolism, cysteine and methionine metabolism, glucosinolate biosynthesis, lysine biosynthesis, folate biosynthesis, and many others. Conversely, 36.36% of metabolic pathways were down-regulated, including arachidonic acid metabolism, sphingolipid metabolism, arginine and proline metabolism, and various biosynthetic pathways like steroid hormone biosynthesis, fatty acid biosynthesis, and purine metabolism (Figure 6D).

### 3.5 Coordinated changes of root-related bacterial community and exudates

**Figure 7:**
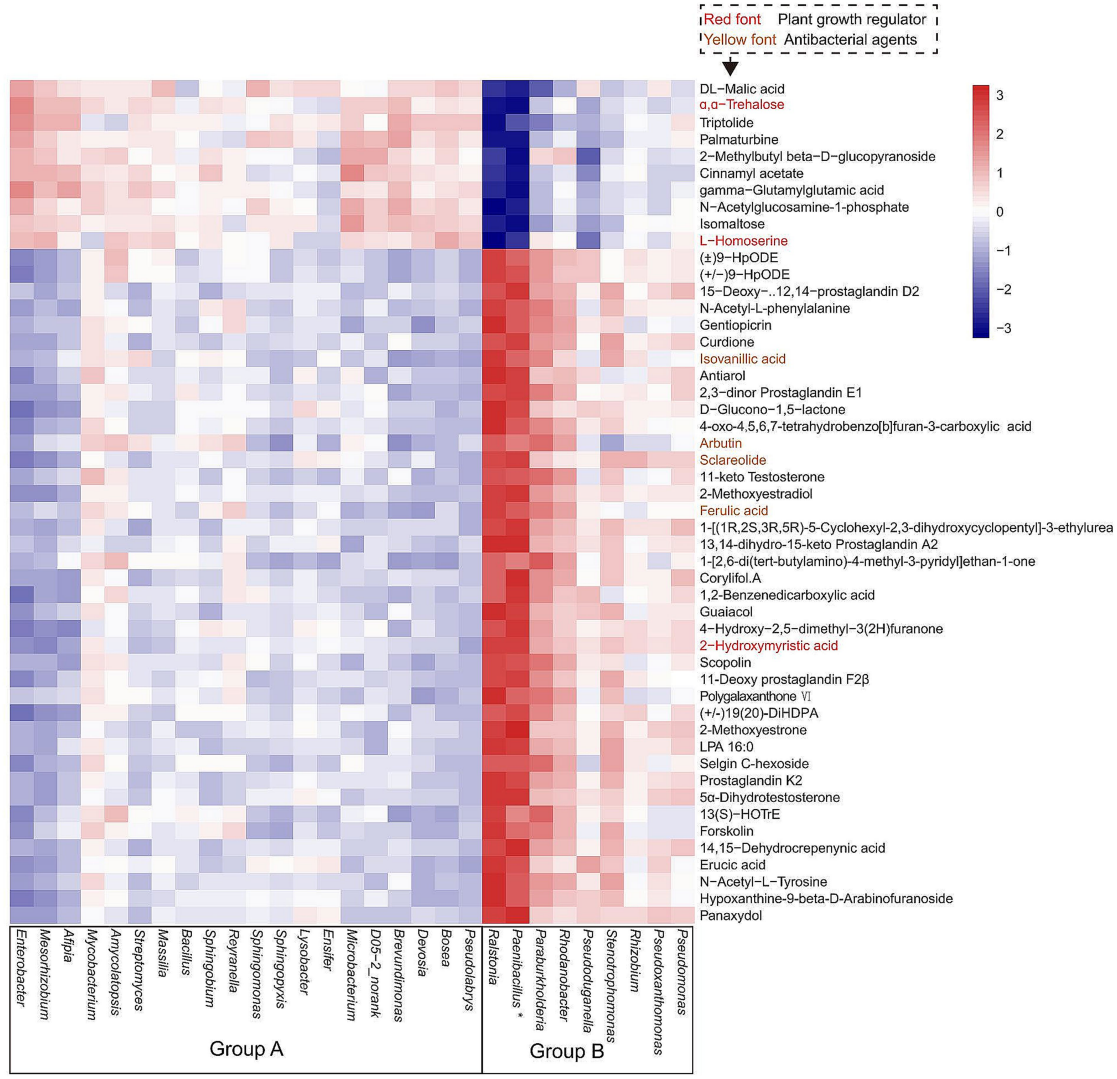
Heat Map illustrating significantly different bacterial genera and exudates.

Root exudates play a crucial role in shaping the root-related bacterial community. Therefore, exploring the relationships between bacterial community and root exudates is of great interest. The Mantel test indicated a significant correlation between the bacterial community in both the rhizosphere and endosphere with root exudates in ginger root (Mantel’s r = 0.15, *p* = 0.04 for rhizosphere bacteria and Mantel’s r = 0.25, *p* = 0.02 for endosphere bacteria). To visually compare the impact of antagonistic endophytes, a heat map analysis was conducted, focusing on bacteria and root exudates correlation with r > 0.4 and *p* < 0.05 (Figure 7). The bacteria were divided into two groups: Group A showed a positive correlation with root exudates up-regulated in T treatment, and Group B exhibited a positive correlation with root exudates down-regulated in T treatment (Figure 7). In Group A, beneficial bacteria such as *Enterobacterium*, *Streptomyces*, and *Bacillus*, whose abundance is positively associated with plant growth regulators like *α*,α-Trehalose and L-Homoserine (highlighted in red font in Figure 7) with AEs addition, were observed. Mantel test also evaluated relationships between Group A and Plant growth regulators, such as α,α-Trehalose (Mantel’s r = 0.28, *p* = 0.001) and 2-Hydroxymyristic acid (Mantel’s r = 0.36, *p* = 0.001) (Table 1). Conversely, in Group B, bacteria such as *Ralstonia*, *Paenibacillus*, and *Stenotrophomonas*, which were positively correlated with antibacterial agents such as Isovanillic acid, Arbutin, Sclareolide, and Ferulic acid (highlighted in yellow font in Figure 7) (Table 1), were identified. Notably, the abundance variation of *Paenibacillus* closely mirrored that of *Ralstonia* (Figure 7).

**Table 1:** Mantel tests of root exudates against endosphere bacteria in Group A and Group B.

Root exudates		GroupA		GroupB	
		*r*	*p*	*r*	*p*
Plant growth regulator	α,*α*-Trehalose	0.279	**0.001**	0.060	0.169
	L-Homoserine	0.068	0.130	0.224	**0.012**
	2-Hydroxymyristic acid	0.356	**0.001**	0.343	**0.002**
Antibacterial agents	Isovanillic acid	−0.032	0.680	0.032	0.348
	Arbutin	−0.050	0.790	0.062	0.269
	Sclareolide	0.056	0.159	0.220	**0.034**
	Ferulic acid	−0.083	0.526	0.022	0.381

Bold font indicates *p* < 0.05.

### 3.6 Interactions between antagonistic bacteria and plant growth

**Figure 8:**
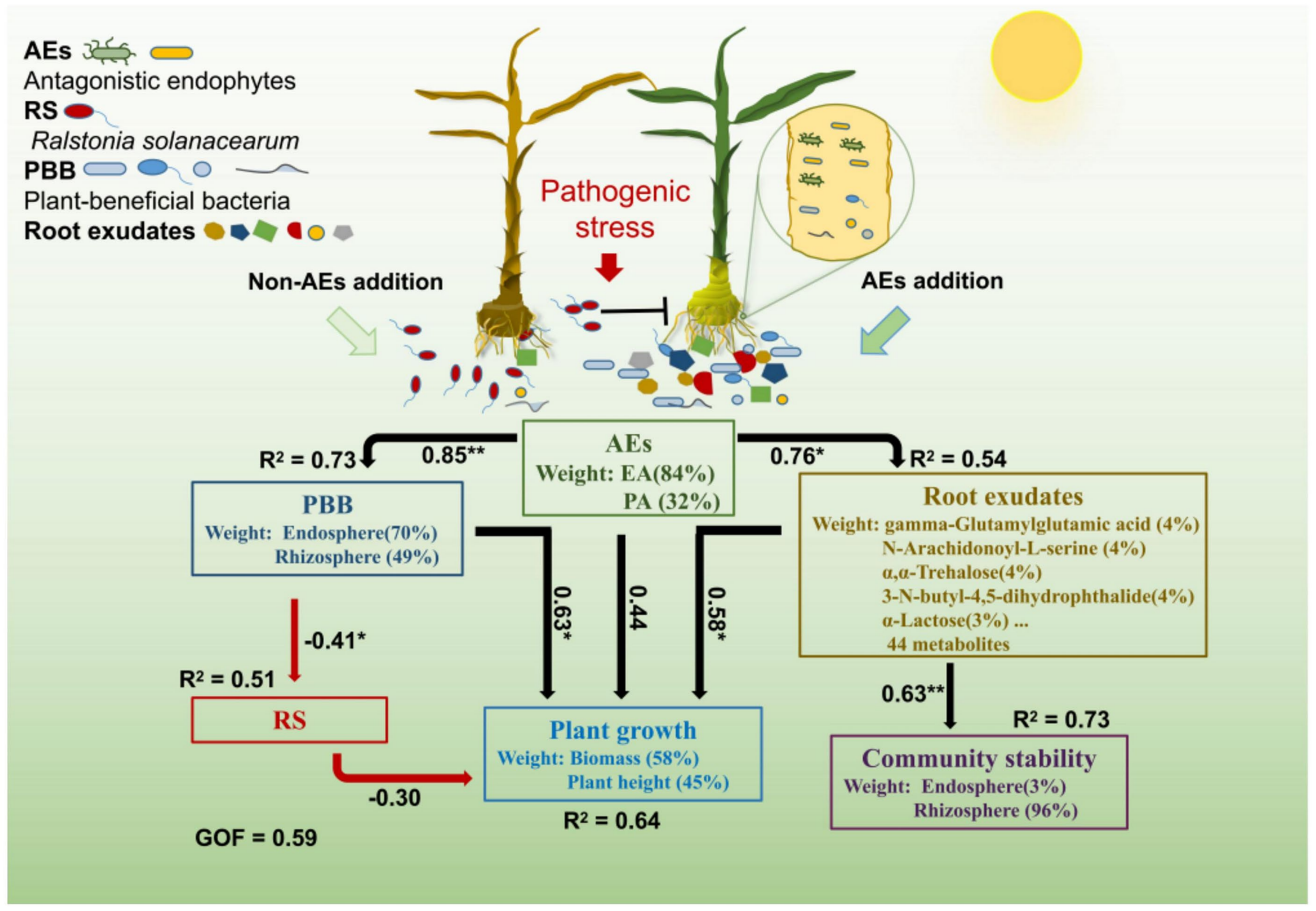
Structural equation model (SEM) comparing the direct and indirect effects of AEs on plant health (plant growth and root colonization of *R. solanacearum*). Black arrows indicate significant positive relationship between the tested variables, while red arrows indicate significant negative relationship retained in the model (non-significant correlations are not shown). The numbers inside the arrows indicate standardized correlation coefficients. GOF (Goodness of Fit), measures the degree of fit between observed variables and the model. **p < 0.01; *p < 0.05. The colonization capacity of AEs and RS in ginger roots was showed in Supplementary Figures S2, S3.

Structural equation model (SEM) analysis and path-modeling estimation were used to disentangle the important interactions behind successful AEs biocontrol outcomes in ginger. Specifically, we explored whether the plant health was directly driven by AEs, or indirectly via shifts in the root-related bacteria and exudates (Figure 8). The final full model had a reasonable fit explaining 51% of the variation in RS density, and 64% of the variation in plant growth, and Goodness of Fit (GOF) = 0.59. We found that addition of AEs did not explain the reduction of RS colonization and plant growth directly (*p* > 0.05). Instead, AEs had strong effects on the PBB (*p* < 0.01) and root exudates (*p* < 0.05), which in turn were correlated with RS density and bacterial community stability, respectively. Interestingly, PBB and root exudates had direct positive effects on plant growth (*p* < 0.05). In addition, root exudates had direct positive effects on bacterial community stability, which explaining 73% of the variation in it. This suggests that AEs mediated the plant health by changing the abundance of PBB and root exudates.

## 4 Discussion

### 4.1 The bacterial community within the root endosphere exhibits a more pronounced positive response to the introduction of antagonistic endophytes compared to the bacterial community in the rhizosphere

Bacterial communities in plant rhizosphere and root endosphere impact the health and nutrition of the host plant (Edwards et al., 2018). Research finds that endophytic bacterial communities are generally more tightly linked to plant performance than rhizosphere bacterial communities (Fitzpatrick et al., 2018; Hannula et al., 2021). In this study, root endophytes exhibited greater sensitivity to the introduction of AEs compared to rhizobacteria. This is primarily evidenced by the more significant changes observed in the endophyte community following treatment with AEs. Unlike rhizosphere bacteria, AEs appear to stimulate root endophytes to produce a higher abundance of beneficial bacteria. Following the introduction of AEs, beneficial bacteria, including *Amycolatopsis*, *Bradyphizobium*, *Devosia*, *Ensifer*, *Flavobacterium*, and *Rhodanobacter*, exhibited a significant increase in abundance within the roots. In contrast, their relative abundance in the rhizosphere remained relatively unchanged. Furthermore, variations in root exudates were found to be closely associated with endophytes, while showing no significant correlation with the rhizosphere bacterial communities. These findings suggest that endophytes respond more favorably to stimulation by AEs. Root endophytes exhibited a more pronounced response to stimulation, a finding that aligns with results from other studies (Fitzpatrick et al., 2018; Hannula et al., 2021). Under drought conditions, the endophytic community associated with rice roots demonstrated a more significant response compared to the rhizosphere community. This response was characterized by a notable increase in monolayer bacterial species, including *Actinomycetes*, *Chlorobacteria*, and *Aerobic Firmicutes* (Hannula et al., 2021).

### 4.2 Antagonistic endophytes enhances the abundance of beneficial bacteria and modifies root exudates

This study observed that the addition of antagonistic endophytes increases the abundance of potential beneficial bacteria, including *Mesorhizobium*, *Massilia*, *Bosea*, and *Methylovirgula*, in both the rhizosphere and endosphere. These diverse groups of bacteria are essential for aiding plants in resisting pathogens, mitigating stress, and enhanceing nutrient absorption. For example, *Mesorhizobium* is known for its involvement in nitrogen conversion (Brauer et al., 2015). *Bosea*, identified as ‘pathogen antagonists’, play a critical role in suppressing the growth of pathogenic *Ralstonia* (Tao et al., 2022). Moreover, strains of *Massilia* genus possess plant growth-promoting properties, such as auxin or siderophore production (Rangjaroen et al., 2017). Overall, antagonistic bacteria contribute to the enrichment of plant-beneficial strains in the root microbiome. It is hypothesized that antagonistic bacteria may assist ginger plants in recruiting beneficial bacteria for their growth and development.

Root exudates are a complex chemical mixture, exhibits spatial and temporal dynamics and is increasingly recognized for its role in plant disease resistance (Canarini et al., 2019). Research suggests that certain ‘disease resistant’ root exudates can inhibit pathogen growth, while others may facilitate pathogenic bacteria colonization (Yuan et al., 2018; Zhang et al., 2020). In this study, various types of root exudates were found to be up-regulated with the addition of AEs. Interesting, many of these exudates have both plant growth promoting and pathogen resistance properties. For instance, gibberellin acts as an antagonist in plant growth and development (Dai and Xue, 2010). Trehalose, a significant form of carbohydrates stored by bacteria or produced by plants, is believed to play a crucial role in controlling carbon allocation in plants to help them cope with stress (Ramon and Rolland, 2007; Krishna et al., 2022). The addition of AEs significantly increased the secretion of trehalose, potentially aiding ginger in enhancing carbon fixation and stress alleviation. Secondary metabolites such as Neohesperidin and demethoxycurcumin, utilized by bacteria to scavenge free radicals, are involved in various physiological activities and are thought to assist plants in overcoming stress (Cesco et al., 2010). Of course, the control group showed an up-regulation of antibacterial metabolites in ginger, which may be a stress response of roots under pathogen stress. Evidently, the up-regulation of root exudates play a vital role in promoting plant growth and development, aiding plants in self-damage repair, and further highlighting the significant contribution of antagonistic bacteria.

### 4.3 Antagonistic endophytes alter the bacteria community by affecting niche breadth and stability

Niche breadth refers to the range of resources utilized by different species within a community. Typically, a broader niche breadth indicates stronger competitiveness, which is advantageous for species coexistence within a community (Konopka et al., 2015). Our study found that the introduction of AEs expanded the niche breadth of rhizosphere bacteria, suggesting an increase in the number of taxa present in the rhizosphere and a higher likelihood of the same taxon being found at different sites (Okie et al., 2015). However, in the endosphere, the niche breadth of generalist species in the control group was wider than that in the group treated with AEs. This suggests that the addition of AEs led to a higher degree of specialization, which may be a disadvantage in resource competition. It is believed that the reduction in niche width in the roots is a trade-off made by specialized species to resist disease invasion.

The robustness of a network was determined by its natural connectivity (Wu et al., 2010). Higher natural connectivity values indicated greater network robustness, reflecting increased competition among bacteria (Coyte et al., 2015). Theoretically, bacterial communities with higher interspecific competition are expected to exhibit greater network stability (Ratzke et al., 2020; Loreau et al., 2021). Our findings showed that soil bacterial networks in the rhizosphere, when supplemented with antagonistic bacteria, displayed higher natural connectivity values, indicating enhanced stability and anti-interference ability. Notably, the strong stability of the bacterial community may hinder extensive colonization of *R. solanacearum* in the rhizosphere due to its anti-interference ability. In the case of the endosphere bacterial community, the natural connectivity of the control group was more stable compared to the group with antagonistic bacteria, with less than 60% of nodes removed. However, this trend reversed when more than 60% of nodes were removed, suggesting that the influx of antagonistic bacteria into the roots led to a simpler bacterial community structure and weakened root stability. Despite the decrease in root bacterial community stability, the colonization of RS significantly decreased, possibly as a trade-off to prevent disease.

### 4.4 Antagonistic bacteria contribute to the health of ginger plants by impacting root-related beneficial bacteria and exudates

The introduction of AEs disrupts the root microenvironment, affecting both biological and abiotic factors. Our comprehensive findings illustrate that the presence of antagonistic bacteria positively impacts ginger plants (Figure 7). AEs contribute to reducing the colonization of RS, lowering the incidence of ginger blast, and enhancing plant growth (Supplementary Figure S1; Figure 2). Therefore, it is essential to elucidate the direct and indirect factors responsible for the beneficial effects of AEs on plants. SEM analysis suggests that the addition of AEs directly enriched the beneficial bacterial taxa and modifies root exudates in the root micro environment. Importantly, these enriched bacterial taxa and up-regulated root exudates significantly boost plant growth. Previous studies have indicated that certain beneficial bacterial taxa can aid plants in resisting pathogens by providing nutrients, enhancing plant tolerance, or inhibiting pathogenic bacteria (Tao et al., 2022). In this research, AEs promote the accumulation of beneficial bacteria in the root zone, directly impacting plant growth and inhibiting pathogens like RS. Similarly, the increased levels of root exudates contribute to plant growth and health, as demonstrated by previous research (Dai and Xue, 2010; Ramon and Rolland, 2007; Krishna et al., 2022). Furthermore, root exudates play a crucial role in maintaining the stability of root-related bacterial communities and promoting plant growth. Rather than targeting individual bacteria, root exudates generally influence the overall structure of bacterial communities (Ulbrich et al., 2022). In a word, the health and growth of ginger plants are enhanced through the enrichment of root-related beneficial bacteria and exudates stimulated by the addition of AEs.

Finally, this study investigated the positive impacts and influencing factors of introducing antagonistic endophytes on ginger growth and health. However, several limitations should be acknowledged. Firstly, the effectiveness of the two antagonistic bacteria was not evaluated independently. Secondly, the alteration of relevant resistance genes/gene clusters in ginger was not determined, leading to a potential lack of comprehensive validation of the plant’s defense mechanisms, indicating a need for further research to address these issues.

## 5 Conclusion

Our research found that antagonistic endophytes have a significant impact on the bacterial community in both the rhizosphere and endosphere of ginger. It recruited PBB and exhibited a negative correlation with the amount of *Ralstonia solanacearum* in soil and suppressed bacterial wilt. Furthermore, antagonistic endophytes enhanced community stability, reducing the impact of pathogens on the overall structure. The application of antagonistic endophytes also stimulated the production of plant growth regulators such as *α*,α-Trehalose and L-Homoserine. These results provide evidence for the efficacy of antagonistic endophytes in pathogen control and optimization of the microenvironment surrounding plant roots.

## Data Availability

The datasets presented in this study can be found in online repositories. The names of the repository/repositories and accession number(s) can be found below: https://ngdc.cncb.ac.cn/omix/preview/lAThuaZF, OMIX017135; https://ngdc.cncb.ac.cn/omix/preview/4Oz8LF8w, OMIX017137.

## Footnotes

1
https://www.nature.com/articles/s43016-023-00869-9#additional-information
