# Supplementary material for: Antagonistic endophytes rescue ginger from bacterial wilt by enriching the beneficial root-associated bacteria and regulating antibacterial root exudates
Source: Front Microbiol. 2026 Jul 30;17:1890218. doi: 10.3389/fmicb.2026.1890218 (PMC13469452; doi:10.3389/fmicb.2026.1890218)
Supplement: Supplementary file 1 [file Data_Sheet_1.docx]

Supplementary Material

# 1 Supplementary Figures and Tables

Identification of antagonistic endophytes (Fig. S1); Relative abundance of rhizobacteria and endobacteria at genus level (Fig. S2); Colonization of two antagonistic endophytic bacteria (PA2457 and PA16) in ginger roots (Fig. S3). Table S1, Physiological and biochemical characteristics of the antagonistic endophytes.

## 1.1 Supplementary Figures

Fig. S1

Fig. S2

Fig. S3

## 1.2 Supplementary tables

Table S1

| Characteristics | PA16 | PA2457 |
| --- | --- | --- |
| Voges-Proskauer test | − | − |
| Methyl red test | + | − |
| Ornithine decarboxylase | + | − |
| Lysine decarboxylase | + | + |
| Oxidase | − | + |
| Gelaune liquefaction | + | + |
| Indole test | − | − |
| D−xylose | + | + |
| L−Arabinose | + | + |
| Lactose | + | − |
| Sorbitol | + | − |
| Sucrose | + | + |
| Citrate | + | + |
| Hydrogen sulfide | − | − |

**Notes**: “＋”, Positive; “－”, Negative.
